# The National Advisory Committee on Sexually Transmitted and Blood-Borne Infections (NAC-STBBI) Statement: Chlamydia and Gonorrhea screening recommendations for non-pregnant adults and adolescents

**DOI:** 10.14745/ccdr.v52i04a07

**Published:** 2026-04-30

**Authors:** Housne Begum, Dominique Basque, Holly Sullivan, Michelle Haavaldsrud, Jennifer Gratrix, Petra Smycek, Annie-Claude Labbé, Stephan Gadient, Annie Fleurant-Ceelen

**Affiliations:** 1 National Advisory Committee on Sexually Transmitted and Blood-Borne Infections Secretariat, Public Health Agency of Canada, Ottawa, ON; 2 National Advisory Committee on Sexually Transmitted and Blood-Borne Infections Chlamydia and Gonorrhea Screening Guideline Project Working Group, Public Health Agency of Canada, Ottawa, ON

**Keywords:** chlamydia, gonorrhea, screening recommendations, non-pregnant adults/adolescents

## Abstract

**Background:**

The National Advisory Committee on Sexually Transmitted and Blood-Borne Infections (NAC-STBBI) undertook a review to adopt, or adapt, the 2021 recommendation for chlamydia and gonorrhea screening issued by the Canadian Task Force on Preventive Health Care (CTFPHC).

**Methods:**

The NAC-STBBI adapted the guideline following the 2014 World Health Organization (WHO) handbook. The Grading of Recommendations, Assessment, Development and Evaluation (GRADE) and GRADE-ADOLOPMENT methods were also applied to determine the certainty of evidence and strength of the recommendations. The guideline question was “should any screening vs no screening/usual care/any other screening be used for non-pregnant people?”. Conflicts of interest were managed according to the Public Health Agency of Canada guidelines.

**Results:**

An environmental scan found 17 guidelines published between 2015 and 2023. Five systematic reviews and 32 original articles were identified and included, addressing screening types, patient values, preferences, feasibility, equity and cost and cost-effectiveness of chlamydia and gonorrhea screening. The search was conducted between October 1, 2019, to May 19, 2023. The certainty of evidence was very low.

**Conclusion:**

The NAC-STBBI suggests universal annual screening for *Chlamydia trachomatis* and *Neisseria gonorrhoeae* infections in all sexually active persons under the age of 30 years (conditional recommendation; very low-certainty evidence). The NAC-STBBI also suggests screening every three to six months for adults and adolescents with multiple sexual partners or a new partner since last tested and “opt-out” screening as frequently as every three months for high prevalence populations or communities (conditional recommendation; very low-certainty evidence).

## Introduction

*Chlamydia trachomatis* (CT) and *Neisseria gonorrhoeae* (NG) were the two most frequently reported bacterial sexually transmitted infections (STIs) in Canada in 2021, with rates increasing steadily over the previous decade. Between 2011 and 2019, rates of CT and NG increased by 26% and 171%, respectively ((1)). An exception to this trend was observed in 2020 and 2021 during the COVID-19 pandemic, where rates of CT and NG decreased due to changes in the demand for, and a lack of access to, STI-related services across Canada ((2)). In 2021, the national rate of reported CT cases was 273.2 per 100,000 population, with the highest rates among females aged 15–29 years old and males aged 20–29 years old. The 2021 nationally reported rate of NG cases was 84.2 per 100,000 population, with the highest rates among females aged 15–29 years old and males aged 20–39 years old ((3)). Although many CT and NG infections are asymptomatic, untreated infections can lead to serious complications, such as chronic pelvic pain, pelvic inflammatory disease (PID), infertility, ectopic pregnancy, epididymo-orchitis and reactive arthritis. Screening is an approach used to detect and treat asymptomatic infections, prevent complications, and reduce transmission. Although findings on the impact of screening on prevalence rates are mixed ((4)), evidence suggests that screening is effective in increasing testing rates of CT and NG and reducing healthcare costs ((5,6)).

*Chlamydia trachomatis* and NG screening is a critical component of an effective STI control program ((7)). Screening for these infections is beneficial in that it stops the spread of infection ((8)), prevents serious complications ((9,10)), and helps an individual maintain good sexual and reproductive health ((11)). However, potential negative effects and the necessity of a universal screening program have been called into question. Firstly, some individuals may experience harms of screening, such as stigmatization and anxiety ((12)). Nonetheless, research shows that young adults would still accept screening despite these concerns ((12)). Secondly, spontaneous clearance of CT and NG infections has been observed in some cases ((13,14)), and is a potential argument against the necessity of a universal screening program for asymptomatic individuals. Given that factors such as bacterial load are associated with the odds of clearance, and that infection transmission can still occur, the possibility of clearance should not hinder screening efforts ((13,14)). Ultimately, screening programs should be implemented if the benefits outweigh the harms and resource use is justifiable.

The main rationale for CT and NG screening is to detect asymptomatic infections in females before they cause PID or other serious reproductive complications ((15)). As well, gay, bisexual and other men who have sex with men (GBMSM) individuals may be at increased risk due to asymptomatic CT and NG infections, especially at the rectal and pharyngeal sites ((16)). The National Advisory Committee on Sexually Transmitted and Blood-Borne Infections (NAC-STBBI) defines screening as a process aimed at detecting a condition in an asymptomatic person. There are several approaches to screening: universal (screening in all sexually active persons with a new or multiple partners, and/or upon request of the individual) ((17)); opportunistic (offering screening when an individual accesses health services and has not undergone recent STBBI testing) ((18)); targeted (screening based on a characteristic associated with increased risk of the condition being detected) ((19)) and systematic (as part of a program in which invitations for screening are sent to all eligible participants and then evaluated for uptake and results) ((20)).

Many countries are assessing their CT and NG screening programs to ensure they are based on the best available evidence. For example, in 2021, the National Chlamydia Screening Programme in the United Kingdom narrowed its focus to only offer screening to females in order to prevent serious consequences of untreated CT infection, rather than focusing on the transmission of infections ((18)). In Australia, universal screening for CT and NG was expanded to individuals aged 15 to 29 due to the higher rates of infections in that age range ((19)).

This guideline focuses on CT and NG screening for asymptomatic sexually active non-pregnant adults and adolescents. In 2021, the Canadian Task Force on Preventive Health Care (CTFPHC) published a guideline on screening for CT and NG in primary care for individuals not known to be at high risk of infection ((20)). The CTFPHC is an independent panel of health professionals that develops guidelines for primary care practitioners, along with related tools and resources, with the aim of improving the health of Canadians ((21)).

The 2021 CTFPHC guideline recommendation to screen for CT and NG differed from the existing screening recommendations issued by the Public Health Agency of Canada (PHAC) ((22)). In response, the NAC-STBBI initiated a review of the guideline. The NAC-STBBI provides PHAC with ongoing, timely advice and recommendations for the development of public health guidance related to STBBI, in support of its mandate to prevent and control infectious diseases in Canada ((23)). The PHAC CT and NG screening recommendations that existed at the time of the 2021 CTFPHC guideline publication were developed in 2010. Key areas of difference between the 2021 CTFPHC and the 2010 PHAC screening recommendations were the types of screening methods, ways to implement the screening, the age group to be screened, and the methodology used to develop the recommendations. The PHAC’s 2010 recommendations were to screen for CT and NG in sexually active individuals younger than 25 years of age and in GBMSM and transgender populations, regardless of age; and targeted screening (screening and repeat screening as indicated) for individuals 25 years of age and older with risk factors for infection ((22)). The PHAC’s 2010 recommendations for screening for CT and NG were based on expert opinion, which was informed by a review of the epidemiological data and scientific literature at the time. In contrast, the 2021 CTFPHC recommendation is annual, opportunistic screening for CT and NG in sexually active individuals younger than 30 years of age with no known risk factors for infection, at primary care visits, using a self- or clinician-collected sample. The 2021 CTFPHC recommendation for screening for CT and NG was developed using the Grading of Recommendations, Assessment, Development and Evaluation (GRADE) approach ((24)), which provides an assessment of the strength of their recommendation as conditional and an assessment of the certainty of the evidence as very low ((20)).

Using the GRADE-ADOLOPMENT method ((25)), the NAC-STBBI CT and NG Screening Working Group (WG) undertook a review of the evidence that informed the 2021 CTFPHC guideline and decided to adapt it to develop a NAC-STBBI guideline on screening for CT and NG. The NAC-STBBI guideline question was, “should any screening vs no screening/usual care/any other screening be used for non-pregnant people?” As part of this process, the NAC-STBBI WG updated the CTFPHC’s systematic review (SR), “Screening for chlamydia and/or gonorrhea in primary health care on effectiveness and patient preferences” ((26)), to examine the most recent evidence on the effectiveness of any screening vs no screening/usual care/any other screening in non-pregnant people.

The objectives of this project were to revise the existing PHAC CT and NG screening guideline and assess new evidence for screening in non-pregnant, sexually active individuals and update, as required, the existing guideline while considering the following recommendations from the CTFPHC: i) increasing the screening age from younger than 25 years to younger than 30 years, regardless of the presence of risk factors for infection other than age; and ii) using an opportunistic approach to screening. This guideline is intended to be used by primary care providers (i.e., nurses, physicians), provincial/territorial sexual health program staff, local public health agencies, sexual health clinics, professional associations, and researchers.

## Methods

The NAC-STBBI CT and NG screening recommendations were developed following the methods outlined in the 2014 edition of the World Health Organization (WHO) handbook for guideline development ((27)). The evidence was assessed using the GRADE approach to determine the certainty of evidence and the strength of the recommendations ((24)). The GRADE-ADOLOPMENT method ((25)), was applied to the 2021 CTFPHC guideline to develop the NAC-STBBI guideline and resulted in an update of the SR and other evidence that informed the guideline, including national case rates and an environmental scan of national and international CT and NG screening guidelines. The GRADE-ADOLOPMENT method is an approach to guideline development that combines adoption, adaptation and, as needed, *de novo* development of recommendations. It emphasizes the importance of using existing guidelines and tailoring them to local needs ((25)). The Appraisal of Guidelines for Research & Evaluation (AGREE) II Instrument was used to evaluate the methodological quality of the identified guidelines ((28)). Finally, PROGRESS-Plus equity factors were identified in the guidelines to assess the range of social determinants and factors that contribute to health equity ((29)). Research implications were also developed by the NAC-STBBI WG to describe the practical applications of the findings. The full CT-NG screening guideline received PHAC approval and was subsequently published on the Government of Canada website.

### Working group

The NAC-STBBI consists of STBBI subject-matter experts, clinicians, researchers, and program managers. The NAC-STBBI established a WG for guideline development consisting of three members of the NAC-STBBI who were supported by the NAC-STBBI Secretariat and other research professionals within PHAC (PHAC team). The PHAC team independently conducted an SR update of available studies, including primary studies and SR on CT and NG screening, and scanned for published screening guidelines by examining the SR review in 2021 by CTFPHC ((20)), and searching the websites of international organizations and provincial/territorial organizations.

### Review of the evidence

The key questions from the SR completed by the CTFPHC were modified and approved by the WG to guide the development of the screening recommendations. More specifically, the population eligibility criteria were expanded to include sexually active individuals younger than 30 years and opportunistic screening was added as an intervention of interest.

**Figure 1:**
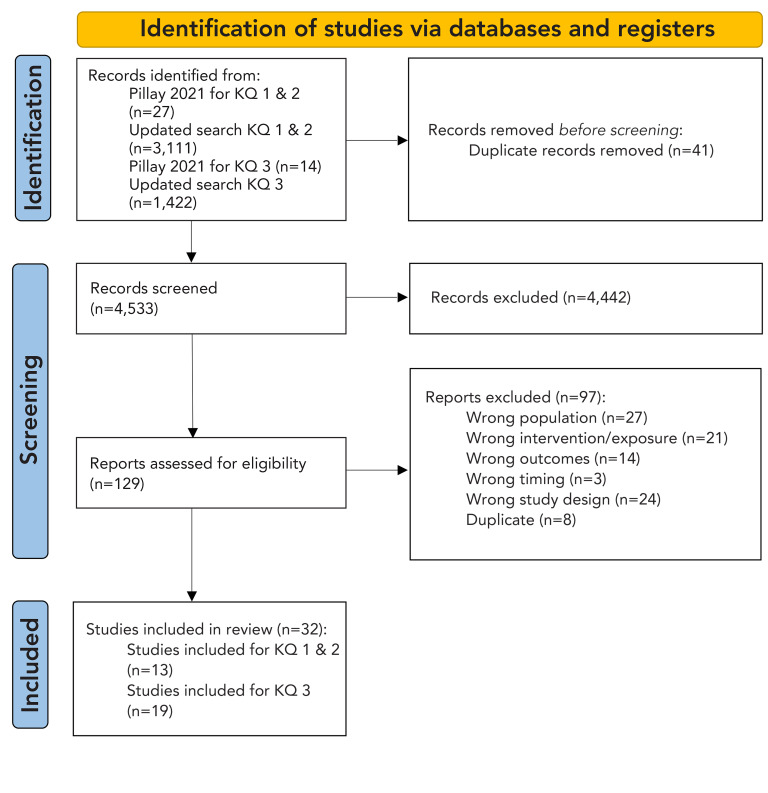
Flow diagram of study selection on Gonorrhea and Chlamydia screening since 2019 Abbreviation: KQ, key question

A hierarchical approach was used to search for evidence to update the CTFPHC SR ((26)). The updated search was conducted for October 1, 2019 to May 19, 2023, using the same search strategies as the CTFPHC SR. The studies included in the original SR were screened against the new eligibility criteria ((26)), and reported on CT and NG screening types, patient values and preferences, equity, feasibility, acceptability, cost and cost-effectiveness analyses. Studies published in English and French were included. The PHAC team also searched for SR, followed by primary studies, when no SRs were available. The grey literature search included searching sources identified in the CTFPHC SR, as well as additional sources identified by the NAC-STBBI WG and Secretariat. Sources searched included trial registries, conference abstracts, reports and CT/NG screening guidelines from international, provincial and territorial public health organization websites. Reference lists of all included studies and relevant SRs that were identified in the updated search were searched by hand for any missed studies. Two members of the PHAC team screened studies, extracted and analyzed the data, and assessed the quality/certainty of the evidence using the GRADE approach (refer to **Figure 1** for the flow diagram of study selection) ((24)). Finally, an environmental scan ((30)) was executed by performing a Google search, which found 17 public health organizations with guidelines on CT and NG screening published between 2015 and 2023; of those, nine were international ((16,31–40)) and eight were Canadian ((20,41–48)).

The certainty of the evidence was assessed at four levels ((24,49)):

· **High:** We are very confident that the true effect lies close to that of the estimate of the effect.

· **Moderate:** We are moderately confident in the effect estimate; the true effect is likely to be close to the estimate of the effect, but there is a possibility that it is substantially different.

· **Low:** Our confidence in the effect estimate is limited; the true effect may be substantially different from the estimate of the effect.

· **Very low:** We have very little confidence in the effect estimate; the true effect is likely to be substantially different from the estimate of effect.

### Management of conflicts of interest

Members of the WGs and NAC-STBBI are required to identify affiliations and conflicts of interest on an annual basis. The NAC-STBBI Secretariat reviews WGs and committee member affiliations to ensure there are no conflicts of interest. The WG and committee members are also asked to identify any new affiliations at the start of every meeting and teleconference. No conflicts were identified by the WG or NAC-STBBI members that would prevent them from participating in the discussion and voting on the committee recommendation.

## Results

### Evidence synthesis

Details of evidence from different sources/types (evidence profile) and evidence to decision judgements are available in **Table 1** and **Table 2**. In addition to the 41 records identified from Pillay *et al.* ((26)), 4,533 records were located from databases. Following the title and abstract screening, 4,442 records were excluded, and 129 records were sought for retrieval. All records were assessed for eligibility and 97 records were excluded (Figure 1). A total of 32 records were included in the review, including original articles on screening types, patient values, preferences, and feasibility and impact on health equity of CT and NG screening. The excluded studies contained the incorrect population, intervention/exposure, outcome, study timing and study design. Five SRs were identified and included ((4,50–53)). The studies from these SRs were assessed for any missing eligible articles that could be included in the evidence review ((4,50–53)). No additional studies were retrieved. Eleven studies were identified for full-text screening from the grey literature search.

**Table 1:** Evidence profile

Key guideline question: Should [any screening 1] vs [no screening/usual care/any screening 2] be used for non-pregnant people?	
**Outcomes by organisms (CT, NG and CT+NG) and screening type**	**Findings/Assessment of certainty of evidence**
**Chlamydia: Universal screening compared to no screening or usual care**	
Chlamydia infection transmission (1 RCT) van den Broek *et al*., 2012	A register based yearly program found chlamydia positivity in the intervention blocks at the first invitation was the same as in the control block/usual care (4.3%) and 0.2% lower at the third invitation than in the control block/usual care, which was not statistically significant (odds ratio: 0.96, 95% CI: 0.83–1.10).
Certainty of evidence	⨁⨁◯◯^a,b,c^ LOW Risk of bias, Indirectness
Chlamydia and pelvic inflammatory disease (PID) (3 RCTs) Oakeshott, 2010; van den Broek *et al.*, 2012; Hocking *et al.*, 2018	Universal screening compared to usual care in primary care did not significantly reduce prevalence of chlamydia in adolescents and young adults. Screening for chlamydia reduces rates of PID; however, the effectiveness of a chlamydia test in preventing PID over 12 months may be overestimated.
Certainty of evidence	⨁◯◯◯^a,b,c^ Very LOW Risk of bias, Indirectness
**Benefits: Gonorrhea screening**	
Nil	Nil
**Chlamydia and gonorrhea: Clinic-based screening compared to no screening or usual care**	
Chlamydia and gonorrhea infection transmission/infection rates (3 studies) Fielder *et al.*, 2013; Reed *et al.*, 2021; Tomcho *et al.*, 2021	In a study comparing clinic-based screening to no screening, 64% of the participants agreed to self-collected vaginal swabs while in clinic with 1% testing positive for an STI. Universal screening compared to usual care in primary care reduced prevalence of chlamydia and gonorrhea in some health clinics including emergency departments but not all.
Certainty of evidence	⨁◯◯◯^a,b,c^ Very LOW Risk of bias, Indirectness
**Chlamydia and gonorrhea: Universal screening compared to no screening**	
Pelvic inflammatory disease	Universal screening compared to no screening for chlamydia and gonorrhea before an IUD insertion did not reduce risk for the infection.
Certainty of evidence	⨁◯◯◯^a,b,c^ Very LOW Risk of bias, Indirectness
**Harms: Chlamydia screening: Any screening versus no screening**	
Negative psychosocial impact (2 studies) Walker *et al.,* 2013; Campbell *et al.*, 2006	One cohort study and a randomized pre-post study examined any screening versus no screening and found that home-based screening and self-collected swabs for chlamydia and gonorrhea have some negative psychosocial impact but is acceptable.
Certainty of evidence	⨁◯◯◯^a,b,c^ Very LOW Risk of bias, Indirectness
**Harms: Gonorrhea screening**	
Nil	Nil
**Harms: Chlamydia and Gonorrhea screening**	
Nil	Nil
**Benefits: Chlamydia screening: Home-based screening compared to clinic-based screening**	
Chlamydia infection transmission (3 studies) Senok *et al.*, 2005; Söderqvist *et al.*, 2020; Gasmelsid *et al.*, 2021	Home-based screening for chlamydia is at least as effective as clinic-based screening in detecting rates of chlamydia. Results for which intervention was greatest was mixed.
Certainty of evidence	⨁⨁◯◯^a,b^ LOW Risk of bias
**Benefits: Gonorrhea screening**	
Nil	Nil
**Benefits: Home-based screening compared to clinic-based screening [health problem and/or population**	
Chlamydia and gonorrhea infection transmission/infection rates (1 study) Reagan *et al.*, 2012	No difference in positivity rates were found between the home-based group compared to the clinic group.
Certainty of evidence	⨁◯◯◯^a,b,d^ Very LOW Risk of bias, Imprecision
**Harms: Chlamydia screening**	
Nil	Nil
**Harms: Gonorrhea screening**	
Nil	Nil
**Harms: Chlamydia and Gonorrhea screening**	
Nil	Nil

Abbreviations: CT, *Chlamydia trachomatis*; CI, confidence interval; IUD, intrauterine device; NG, *Neisseria gonorrhoeae*; PID, pelvic inflammatory disease; RCT, randomized controlled trial; STI, sexually transmitted infection

^a^ Symbols to describe certainty of evidence in evidence profiles: high certainty ⊕⊕⊕⊕, moderate certainty ⊕⊕⊕◯, low certainty ⊕⊕◯ and very low certainty ⊕◯◯◯

^b^ Risk of bias; issue with blinding and the authors did not mention any information related to the use of an appropriate analysis method that adjusted for all the critically important confounding domains

^c^ Interventions do not always directly match the research question

^d^ Total number does not meet the optimum sample size

**Table 2:** Summary of evidence-to-decision framework judgements

Domain	Judgement
Problem	Yes
Desirable effects	Moderate
Undesirable effects	Small
Certainty of evidence	Very low
Values	Possibly important uncertainty or variability
Balance of effects	Favors the intervention
Resources required	Don’t know
Certainty of evidence of required resources	Very low
Cost effectiveness	Probably favors the intervention
Equity	Probably increased
Acceptability	Probably yes
Feasibility	Probably yes

### Summary of the evidence

The certainty of evidence for the screening of CT and NG in asymptomatic, non-pregnant individuals was very low. The review retrieved 32 articles that included five randomized controlled trials ((4,15,50–52)), five cohort studies ((53–57)), seven qualitative studies ((12,58–63)), five cross-sectional studies ((8,64–67)), four cost-effectiveness studies ((9,10,68,69)), a prospective delayed start pragmatic study ((6)), a controlled pre-post quasi experimental study ((70)), a randomised pre-post study ((71)), a retrospective service evaluation ((11)), a mixed-method study ((72)), and a health utility study ((73)).

The environmental scan retrieved 11 guidelines ((16,31–40)) from nine international organizations and nine guidelines ((20,41–48)) from eight Canadian public health organizations on CT and/or NG screening. These guidelines varied mostly on the approach to screening used (opportunistic, universal, or risk-based screening). Most guidelines recommend screening individuals younger than 25 years of age ((16,32–48)), while only two organizations recommend screening individuals younger than 30 years of age ((20,31)).

The WG decided to use indirect evidence from the NAC-STBBI syphilis screening recommendations ((17)) because of a lack of direct evidence on the frequency of CT and NG screening.

### Benefits of screening

The benefits of screening for CT were assessed in three randomized control trials and a controlled cohort study comparing universal screening to usual care. The authors found little evidence that universal screening impacted CT positivity rates ((4)), prevalence rates ((50)), and incidence of PID ((4,38,50,53)). Notably, the results on positivity rates were not consistent. A prospective delayed start pragmatic study and a pre-post quasi experimental study assessing clinic-based universal screening found that rates for CT significantly decreased in primary care (paediatrics clinic and family clinic), and in one of two emergency departments included in the study ((6,70)).

Three studies comparing home-based screening to clinic-based screening on CT infection transmission found that home-based screening, where an asymptomatic individual orders a self-sample test kit from a website, collects their sample and sends it to a laboratory for processing, is beneficial to increase access to services. A randomized controlled trial found that clinic-based opportunistic screening detected slightly higher rates on CT than home-based screening ((52)). However, a retrospective cohort study and a pre-post study reported that non-invasive self-sampling (urine sample or vaginal swab) resulted in a higher CT detection rate, suggesting that at-home screening is at least as effective as clinic-based screening, while also being an accessible alternative to in-clinic screening ((11,55)). In contrast, two studies found that NG rates increased following the implementation of clinic-based universal screening in the family clinic, while decreasing in the pediatric clinic and one of two emergency departments ((6,70)).

### Harms of screening

Three studies found little evidence of negative psychosocial impact from screening efforts. A cohort study comparing universal screening to no screening found that, irrespective of the test result, females were afraid about the possibility of receiving a positive test ((56)). However, females who received a positive test had less concern about their test, their future health and their partners reaction compared to females who reported on how they thought they would feel if they received a positive test ((56)). A randomized pre-post study comparing CT home-based screening to no screening found that the invitation to participate in screening resulted in higher anxiety scores in females compared to males ((71)). Screening did not impact overall well-being, as anxiety decreased following the submission of the test among males, and decreased in females following a negative test result ((71)). Similar findings were reported in a randomized controlled trial investigating screening for CT and NG, where males were more likely to complete screening at home, compared to in-clinic, with no difference in positivity rates ((51)).

### Patients’ preferences and values

Patients’ preferences and values on CT screening were assessed in seven qualitative studies. These studies assessed no screening or compared universal screening to no screening. Most participants reported that they would get tested for CT and would encourage others to get tested ((59)). The negative emotions that arose from screening were related to embarrassment with sexual health issues, the association that STIs have with sexual promiscuity ((12,58,60)), perceptions of public stigma ((56,58,62)), concern for their future reproductive health ((60)), anxiety regarding notifying their partners ((60)), and anonymity ((12)). Despite these potential barriers to testing, participants reported recognizing the need to balance harms of screening with the benefits ((56,57)).

Qualitative studies assessing perceived barriers to CT and NG screening found that fear and aversion ((65)), social stigmas ((62,65)), negative consequences ((62,65)), confidentiality ((62)), and the reputation of the clinic represented significant barriers to being tested, which could increase the risk of spreading infection to others ((62,65)).

Cross-sectional data suggests that both positive and negative beliefs influence the decision to seek regular CT testing. Positive beliefs (such as the reassurance of not being infected), increases the intention to seek CT testing; meanwhile, negative beliefs (such as feeling that getting tested is embarrassing) reduces intentions to seek testing ((64)). Females were significantly more likely to hold positive beliefs than males ((8)).

A randomized controlled trial feasibility study and a retrospective cohort study comparing home-based to clinic-based CT screening found that a higher percentage of individuals who tested at home returned samples compared to those who were subject to clinic-based opportunistic screening ((52,55)). An exception to this finding was among females under 20 years old, who returned more samples in the clinic-based group than the home-based group ((52)). A service evaluation study did not find that online self-sampling for CT increased the number of individuals screened ((11)). Similarly, a controlled trial with randomized stepped wedge implementation did not support a registered based CT screening program as participation rate declined over screening rounds ((4)).

A randomized controlled trial and a retrospective cohort study found that home-based CT screening did not vary in opt-out rate compared to usual-care and opportunistic screening ((52)). Findings on self-sampling were mixed, where a higher proportion of males used self-sampling compared to usual-care or opportunistic screening ((55)); however, females were more likely to take part in self-sampling than males when comparing universal self-sampling to usual care ((4)). Furthermore, participation rates were higher among the older age groups ((4)). Qualitative findings related to acceptability of CT and NG screening found a very high rate of acceptance with the idea of offering universal screening to adolescents using a tablet-based NG/CT screening tool in a private room. Adolescents felt it would address concerns about discussing NG or CT screening with clinicians, while parents or guardians felt that using tablets may increase participation to screening but were concerned about the lack of personal interaction with a healthcare provider ((63)).

### Making recommendations

The NAC-STBBI WG developed the recommendations in seven meetings held between June and September 2024. The WG members reviewed the evidence-to-decision table presented by the PHAC team, and the available national and provincial (Alberta, Québec) epidemiological data ((74,75)). During the formulation of the recommendations, the NAC-STBBI WG considered both the desirable and undesirable outcomes of screening interventions, the values and preferences, feasibility, equity, resources, cost and cost-effectiveness of the interventions. They also discussed the implementation of the recommendations and research gaps. The discussion was facilitated by a methodologist with the goal of reaching consensus across the NAC-STBBI WG.

The draft recommendations, the evidence, and the WG’s rationale for the recommendations were first presented to the NAC-STBBI on June 27, 2024 for their input. With the committee’s suggestions, final recommendations were compiled by the WG and sent to the NAC-STBBI on September 5, 2024 for their review. Consensus and approval of the recommendations was obtained on September 26, 2024. The PHAC approval was provided by the Vice-President of the Infectious Diseases and Vaccination Programs Branch on October 24, 2024. The recommendations were subsequently added to PHAC’s Chlamydia and lymphogranuloma venereum guide ((76)), and Gonorrhea guide ((77)), within the STBBI Guides for Health Professionals ((78)).

According to the GRADE approach ((24)), the certainty of evidence was rated as very low and the strength of the recommendations was rated as conditional. The conditional recommendations are worded as “the NAC-STBBI guideline suggests....”. The implications of conditional recommendations are:

· **For patients:** “The majority of individuals in this situation would want the suggested course of action, but many would not. Decision aids may be useful in helping patients to make decisions consistent with their individual risks, values, and preferences.”

· **For clinicians:** “Different choices will be appropriate for individual patients; clinicians must help each patient arrive at a management decision consistent with his or her values and preferences. Decision aids may be useful in helping individuals to make decisions consistent with their individual risks, values, and preferences.”

· **For policy makers:** “Policy making will require substantial debate and involvement of various stakeholders. Performance measures should assess if decision-making is appropriate.”

· **For researchers:** “The recommendation is likely to be strengthened (for future updates or adaptation) by additional research. An evaluation of the conditions and criteria (and the related judgments, research evidence, and additional considerations) that determined the conditional (rather than strong) recommendation will help identify possible research gaps.”

### Recommendations

Screening is a process aimed at detecting a condition in an asymptomatic person. Recommendations developed by the NAC-STBBI are made at the population-level (**Box 1**). It is important to note that they may not apply to specific individuals, particularly as it relates to individuals in groups or communities who may have higher rates of CT and NG when compared to the general public. It is always essential to consider the context of the risk behaviours and epidemiological factors outlined in the recommendation.

Box 1 **Recommendations**

**Table:** 

**Recommendation 1: *Chlamydia trachomatis*/*Neisseria gonorrhoeae* screening for non-pregnant adults and adolescents** The NAC-STBBI suggests universal annual screening* for *Chlamydia trachomatis* and *Neisseria gonorrhoeae* infections in all sexually active persons under the age of 30 years. **(*Conditional recommendation; very low certainty of evidence*).**
**Recommendation 2: *Chlamydia trachomatis*/*Neisseria gonorrhoeae* screening for adults and adolescents with multiple partners or a new partner** The NAC-STBBI suggests screening* every three to six months for *Chlamydia trachomatis* and *Neisseria gonorrhoeae* infections in all persons with multiple sexual partners or a new partner since last tested. **(*Conditional recommendation; very low ­certainty of evidence*).**
**Recommendation 3: *Chlamydia trachomatis*/*Neisseria gonorrhoeae* screening for high prevalence groups and communities** The NAC-STBBI suggests that “opt-out” screening* for *Chlamydia trachomatis* and *Neisseria gonorrhoeae* infections be considered as frequently as every three months** in populations or communities*** experiencing high prevalence of CT and NG (and other STBBI), such as: · Gay, bisexual and other men who have sex with men (GBMSM) · People living with HIV · People who are or have been incarcerated · People who use substances or access addiction services · Some Indigenous communities **(*Conditional recommendation; very low certainty of evidence*).**
**Considerations** * Options to increase screening uptake should be explored. They include: · Opportunistic screening (offering screening when an individual accesses health services and has not undergone recent STBBI testing) · Increasing accessibility and normalizing testing through strategies such as outreach testing and opt-out screening · Facilitating sample collection through strategies such as non-invasive collection of specimens, including self-sampling ** Consider aligning screening with other health services (“opportunistic screening”) such as HIV or addiction care. *** Consider local epidemiology, travel history and individual patient risk factors when determining which groups/communities to target. Factors associated with *Chlamydia trachomatis/Neisseria gonorrhoeae* infections ((79–81)): **Behaviours/activities** · Sexual activity involving contact with oral, genital or anal mucosa ((81–84)) · Multiple sexual partners ((82–85)) · Sexual contact with a person infected by CT/NG (“known case”) or other STBBI ((84,85)) · Substance use, including chemsex ((84,85)) **Epidemiological/biological** · Previous CT/NG infection or other STBBI ((83,84)) · HIV infection ((82–85)) · High prevalence in geographical area ((82–85)) · High prevalence in population groups ((82–85)) · Housing instability/street involvement ((82,83,86,87))

Abbreviations: CT, *Chlamydia trachomatis*; NAC-STBBI, National Advisory Committee on Sexually Transmitted and Blood-Borne Infections; NG, *Neisseria gonorrhoeae*

## Discussion

To implement this screening recommendation, clinicians are suggested to offer universal annual screening in all sexually active persons under the age of 30 years. For individuals 30 years of age or older, a risk assessment should be conducted in all sexually active individuals, since those who are at high risk for CT and NG infection may not always self-identify or be easily identified.

*Chlamydia trachomatis* and NG infections are often associated with social stigma, shame and embarrassment, which could prevent an individual from seeking screening and treatment ((20,88)). Making STI screening routine has been suggested as a way to help destigmatize STI testing by not singling out individuals because of their reported or assumed risk behaviours ((31)). Making screening integral to care helps to reduce stigma and normalize conversations around sexual health. Individuals who have had a negative experience with the healthcare system may be reluctant to seek care. Alternate strategies and approaches may be needed to enhance trust and improve comfort with accessing health services. These strategies may vary across provinces/territories, local communities, and/or population groups. A “one style fits all” strategy is unlikely to be successful.

Options to increase screening uptake should be explored. For example, opportunistic screening capitalizes on existing healthcare interactions and relations by offering screening when individuals access health services and have not undergone recent STBBI testing ((18)). The CTFPHC recommends opportunistic screening of sexually active individuals younger than 30 years of age for CT and NG at primary care visits ((20)). Grennan *et al.* endorses CTFPHC’s new screening guideline and adds that the benefits will not only increase the number of cases diagnosed, but it will decrease transmission, and possibly reduce the likelihood of being a risk factor for HIV acquisition ((7,89)). Outreach testing (testing in a community-based setting) and opt-out programs (offering testing automatically unless the individual declines) are two strategies that have been shown to increase accessibility and normalize testing. Opt-out screening have demonstrated greater success in identifying cases compared to opt-in programs (offering testing to those who accept) ((66,90)). Other strategies to increase screening are the utilization of self-collection kits, non-invasive collection specimens and home-based screening. Increased availability of point of care testing, self-test and rapid tests offer new ways to test the public and may improve acceptability and uptake ((20,91,92)).

Although the optimal screening interval is unknown, the NAC-STBBI suggests annual screening for non-pregnant adults and adolescents less than 30 years of age, three to six months for all persons with multiple sexual partners or a new partner, and every three months in populations or communities experiencing high prevalence of CT or NG infections may be cost-effective ((68,69,93)).

The PHAC and NAC-STBBI continue to monitor the changes in the epidemiology of high prevalence populations/behaviours. The publication of new evidence and the modification of screening guidelines among health authorities is monitored in order to respond to the latest developments. These screening recommendations will be revised if new evidence becomes available in the coming years, or if the epidemiological situation changes to justify subsequent updates to the recommendations. However, there are still knowledge gaps on the natural history of CT and NG infections. *Neisseria gonorrhoeae* is considered a serious public health threat since it has been increasingly developing resistance to antimicrobial drugs recommended as treatment ((94)). Further research in the potential harms of overdiagnosis of infection that may clear spontaneously and the overuse of antimicrobials which may contribute to antimicrobial resistance is crucial to evaluate if intensive screening and treatment programs are justified.

Above all, STBBI research is mainly focused on specific groups of individuals, such as people living with HIV and GBMSM. Studies focused on the general population are lacking and present a significant gap in evidence. Attempting to generalize evidence from these groups to apply to the general population is not always practical given their significant differences in population groups. Traditionally, GBMSM populations have experienced higher rates of STBBI infections resulting in suggested higher frequency of STBBI screening among this population. Addressing the research gaps listed above would be beneficial to inform whether to update or reaffirm the CT and NG screening recommendations in the future. The focus of future research studies should also be targeted to the serious outcomes of untreated CT and NG infections. In addition, further research comparing different screening intervals would be informative.

### Limitations

There was variation in the certainty of evidence and applicability of studies. Much of the evidence used to inform the development of these recommendations was based on studies conducted on various age ranges. Studies examining CT and NG screening in the group that is less than 25 years old versus the group that is less than 30 years old were lacking. There was also limited evidence on the comparison of different screening programs, such as opportunistic screening, universal screening, self-sampling and targeted screening and whether the interventions are cost-effective. Despite implementing a range of interventions to control CT and NG infections, there is a lack of high certainty evidence that population prevalence can be reduced by screening programs or opportunistic testing. There is also a lack of high-quality empirical evidence for the benefits of testing for the prevention of PID, ectopic pregnancy, infertility and epididymo-orchitis. Since the NAC-STBBI WG did not have a patient representative, patient perspectives were acquired through the evidence. Inclusion was restricted to English and French language studies only.

## Conclusion

*Chlamydia trachomatis* and NG infections have increased steadily over the past few years. Ongoing review and monitoring of the most recent Canadian surveillance data is integral to ensure individuals/populations with high infection prevalence are identified quickly. It is important for healthcare providers to be aware of the growing public health burden of CT and NG infections so that cases can be identified and treated and the onward transmission of the infection interrupted. Overall, the NAC-STBBI suggests three screening recommendations: i) universal annual screening for CT and NG infections for all sexually active persons under the age of 30 years; ii) screening every three to six months for the same infections in individuals with multiple sexual partners or a new partner since last tested; and iii) “Opt-out” screening for CT and NG infections to be considered as frequently as every three months in populations or communities experiencing high prevalence of CT and NG (and other STBBI). The certainty of the evidence for the screening of CT and NG is very low and the strength of the recommendations are conditional.
